# Generalists at the interface: Nematode transmission between wild and domestic ungulates

**DOI:** 10.1016/j.ijppaw.2014.08.001

**Published:** 2014-08-13

**Authors:** Josephine G. Walker, Eric R. Morgan

**Affiliations:** a School of Biological Sciences, University of Bristol, Life Sciences Building, 24, Tyndall Avenue, Bristol BS8 1TQ, UK; b Cabot Institute, University of Bristol, Bristol BS8 1UJ, UK

**Keywords:** Nematode, Ungulate, Parasite, Domestic, Generalist

## Abstract

Many parasitic nematode species are generalists capable of infecting multiple host species. The complex life cycle of nematodes, involving partial development outside of the host, facilitates transmission of these parasites between host species even when there is no direct contact between hosts. Infective nematode larvae persist in the environment, and where grazing or water sources are shared ingestion of parasite larvae deposited by different host species is likely. In this paper we examine the extent to which nematode parasite species have been observed in sympatric wild and domestic ungulates. First, using existing host–parasite databases, we describe expected overlap of 412 nematode species between 76 wild and 8 domestic ungulate host species. Our results indicate that host-specific parasites make up less than half of the nematode parasites infecting any particular ungulate host species. For wild host species, between 14% (for common warthog) and 76% (for mouflon) of parasitic nematode species are shared with domestic species. For domestic host species, between 42% (for horse) and 77% (for llamas/alpacas) of parasitic nematode species are shared with wild species. We also present an index of liability to describe the risk of cross-boundary parasites to each host species. We then examine specific examples from the literature in which transmission of nematode parasites between domestic and wild ungulates is described. However, there are many limitations in the existing data due to geographical bias and certain host species being studied more frequently than others. Although we demonstrate that many species of parasitic nematode are found in both wild and domestic hosts, little work has been done to demonstrate whether transmission is occurring between species or whether similar strains circulate separately. Additional research on cross-species transmission, including the use of models and of genetic methods to define strains, will provide evidence to answer this question.

## Highlights

• Nematodes vary in host range, affecting potential for cross-species transmission.
• Host-specific parasites account for <50% of the parasite species infecting a host.
• Goats are most and horses are least liable to nematodes carried by wildlife.
• Plains zebra and mouflon are most liable to nematodes carried by livestock.
• Existing knowledge is biased, 84% of references are from Africa, Europe, North America.

## Graphical Abstract

**Figure:**
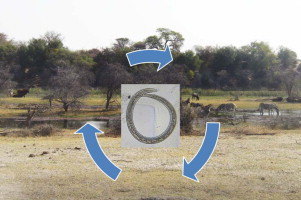

## 1 Introduction

Parasites within the diverse class Nematoda infect a broad range of hosts including humans, domestic and wild animals, and plants (Blaxter et al., 1998). Nematodes are a ubiquitous part of an ecosystem and many are generalist parasites. In this paper we define a generalist parasite as a parasite species which has been found to survive and reproduce in more than one type of host in a given stage of its life cycle. The majority of parasitic nematodes have a multi-phase lifecycle, with a parasitic phase inside the host, and a free-living stage in the environment or intermediate hosts (Taylor et al., 2007). For most nematodes of ungulates, the definitive host is infected trophically, i.e. by ingesting infective larvae on pasture, though in some species larvae burrow through the skin or are transmitted by invertebrate intermediate hosts (Anderson, 2000). Trophic transmission of nematodes involves long-living infective stages and opportunistic exposure to hosts, and is therefore likely to provide an opportunity for infection of alternative host species (host switching).

In many parts of the world grazing land is shared between wild and domestic species, leading to the potential for transmission of parasitic nematodes between these groups. When studying the interactions between wildlife, livestock, and their parasites, it will be important to understand the historical context and patterns of contact and relatedness between the host species. If we are to make predictions of changes in host–parasite interactions due to climate or land use change at the interface between conservation areas and livestock-rearing areas, we must incorporate observations of existing host–parasite relationships with the possibility of host-switching. An extensive discussion of the theory of ecological fitting, whereby parasites possess the adaptations necessary to survive in a new host, and its implications for host-switching and the evolution of parasite generality is beyond the scope of this paper, but has been previously developed (Agosta and Klemens, 2008; Agosta et al., 2010; Hoberg and Brooks, 2008). A better understanding of the extent of and potential for cross-species transmission could influence management strategies for both domestic and wild ruminants in a variety of geographical contexts (Morgan et al., 2005).

Studies of disease transmission at the wildlife/livestock interface rarely discuss nematodes (Bengis et al., 2002; Gortázar et al., 2007; Kock, 2005; Wambwa, 2005). While other multi host diseases, such as foot and mouth disease and brucellosis, cause clear economic impacts on livestock producers, the role of nematodes is more subtle and nematodes on their own are not usually causes of high mortality. Nematodes have, however, been shown to strongly affect health, production, and population dynamics, even if these impacts have not been broadly quantified or generalized. It is difficult to estimate the total clinical impact of nematode infections because clinical signs are non-specific and dependent on the number of parasites in the host (Brooker, 2010). The economic cost of infection and of control of nematodes in the livestock industry is also difficult to estimate, in part because parasites can affect the productive value of animals in several different ways, including reductions in milk production, growth rate, fertility, and susceptibility to other diseases (Cobon and O'Sullivan, 1992; Perry and Randolph, 1999; Thumbi et al., 2013). For example, *Teledorsagia* (formerly known as *Ostertagia*, see Wilson et al., 2004) species contributed to mortality of Soay sheep during a malnutrition-related population crash, but the effect attributable to the parasites was not straightforward to calculate (Gulland, 1992). In wildlife, the seminal example of population-level impacts of nematodes is the regulation of population cycles in red grouse by *Trichostrongylus tenuis* (Hudson et al., 1998). Another gastrointestinal nematode parasite, *Ostertagia gruehneri*, was shown to have an effect on reindeer population density (Albon et al., 2002).

Historically, studies of nematode parasites in wild ruminants have been descriptive accounts rather than clinical, as knowledge of the morbidity and mortality that the parasites cause wild hosts is limited (Hoberg et al., 2001). In addition, few papers directly address transmission between wild and domestic hosts. For example, a long-running series of papers on parasites of domestic and wild animals in South Africa has documented the parasite fauna in different host species, but has not led to a published synthesis of the extent of overlap between parasites of different host species (for example: Boomker et al., 1987; Horak et al., 2004; Van Wyk and Boomker, 2011). However, some regional reviews have been conducted addressing the extent of overlap between nematodes of domestic and wild ungulates, and these examples are discussed in Section 3.3. These accounts, while primarily descriptive, do include limited examples of a clinical or ecological perspective. In addition, while there has been much recent work on ecological theory related to host–parasite interactions, this is only beginning to be applied to the interface between wild and domestic ruminants (Hoberg et al., 2008).

In this paper we examine the extent to which nematode parasite species have been observed in sympatric wild and domestic ungulates globally. First, using existing host–parasite databases, we describe the extent to which parasite species overlap between wild and domestic ungulates is expected. We define a simple index of liability to compare host species by the extent to which their parasite fauna is specialized versus generalized, i.e. crossing the wild–domestic boundary. We then review the literature in which transmission of nematode parasites between domestic and wild ungulates is directly addressed, to demonstrate some of the limitations of current knowledge. The aim is to ascertain general patterns in recorded overlap of nematode fauna between wild and domestic ungulate species, in order to quantify the propensity of cross-transmission to occur in nature.

## 2 Methods

We reviewed known host–parasite associations in wild and domestic ungulates to determine the extent of overlap between nematode parasites in different host species. Nematodes of many wild ruminants have been described in the literature and these references compiled into the Global Mammal Parasite Database (GMPD) (Ezenwa et al., 2006). The GMPD includes references to parasite descriptions in wild artiodactyl and perissodactyl ungulates, including nematodes and other helminths, viruses, bacteria, and arthropods. A version of the database with citations from 1981 to 2002 is available online at www.mammalparasites.org. We used this online database as well as more recent records compiled by P.R. Stevens (unpublished data). Parasite names may be out of date in existing publications, so parasite species names are corrected during entry into GMPD according to current online databases (Nunn and Altizer, 2005). Various veterinary checklists for nematode parasites of domestic animals are available; we extracted these data from *Veterinary Parasitology* (Taylor et al., 2007). From this source, we were able to define the nematodes that parasitize domestic species: cattle, sheep, goats, pigs, horses, camels, and camelids (llamas and alpacas) (Taylor et al., 2007). A list of nematodes infecting donkeys and horses was also included (Matthee et al., 2004).

Records of parasitic nematodes in the GMPD which included a full binomial species name were included for the purposes of this analysis. Subspecies classifications for hosts and parasites were eliminated, and corrected binomial species classifications were used instead. Host–parasite associations which were recorded in the database but where prevalence was reported as zero were excluded.

Title, abstract, and/or full text of each reference was examined to find the geographic location of the study and whether multiple parasite species and/or multiple host species were included. References were categorized by geographical region (Polar, Asia and Pacific, Europe, Latin American and Caribbean, North America, West Asia, Africa) as defined by Global Environment Outlook 3 (GEO-3) of the United Nations Environment Program. A reference was considered to include multiple parasite species if it discussed multiple parasite species or diseases within the same animal. Papers that assessed, discussed, or inferred transmission of nematodes between wildlife and livestock were identified for further review.

Domestic and wild ungulate host–parasite lists were combined and analyzed using R (R Development Core Team, 2009), including the package bipartite (Dormann et al., 2009). For each host, the ‘degree’ was calculated, defined as the number of parasite species infecting that host. The number of references describing each wild host was calculated and correlation with degree was assessed using Spearman's rank correlation (cor.test). The number of parasite species unique to a host species was calculated, as was the number of parasite species shared with the other group (wild/domestic). The remaining parasites are shared with other hosts in the same group.

A simple index, *L*, of liability to parasite generalism was calculated for each host by

$$L=\frac{S_{\text{WD}}-U}{d}$$

where *S*_WD_ is the number of parasites shared with the other group (wild/domestic), *U* is the number of parasites unique to that host, and *d* is the degree, or total number of parasites of that host.

The *L* index is designed to range from −1 (entirely host-specific parasites) to 1 (entirely parasites shared with the other group). This represents the degree to which a host species is vulnerable to infection with generalist parasites, where hosts with entirely host-specific parasites would have an index of −1, while a host with primarily group-specific parasites (or those with half and half host-specific and cross-group) would have an index close to 0, and those with entirely cross-group parasites would have an index of 1.

In order to identify more recent papers describing nematodes transmitted between wild and domestic species which may have been excluded from the database, we conducted systematic searches on the Web of Science database on 29 January 2014 using topic search terms “nematode” and “wildlife” and “livestock” (17 results) as well as topic search terms “nematode” and “wildlife” and “domestic” (53 results). Titles, abstracts, and full text were examined as necessary to determine whether the paper fits the inclusion criteria of specifically addressing the transmission of nematodes in both wild and domestic ungulates. Those papers which were not described in GMPD were not included in the quantitative analysis but are described in Section 3.3.

## 3 Results

### 3.1 References from the GMPD

A total of 241 references published between 1982 and 2009 were included from GMPD. When these were combined with the domestic parasite list, a total of 412 nematode species were reported in 76 wild and 8 domestic host species. These references were highly geographically biased, with 84% of the references describing ungulates in Africa, Europe, or North America (Table 1).

Most references described multiple parasites in a single host species, and only 49/241 (20%) took a multiple host and multiple parasite perspective (Table 2).

### 3.2 Parasitic nematodes of wild and domestic ungulates

Both wild and domestic ungulates host a diverse set of nematode parasites, with 72 different species of nematode reported in the domestic horse, the host with the highest degree (number of parasite species). When all 84 domestic and wild species are ranked by degree, six out of the seven highest degrees are domestic animals.

**Fig. 1:**
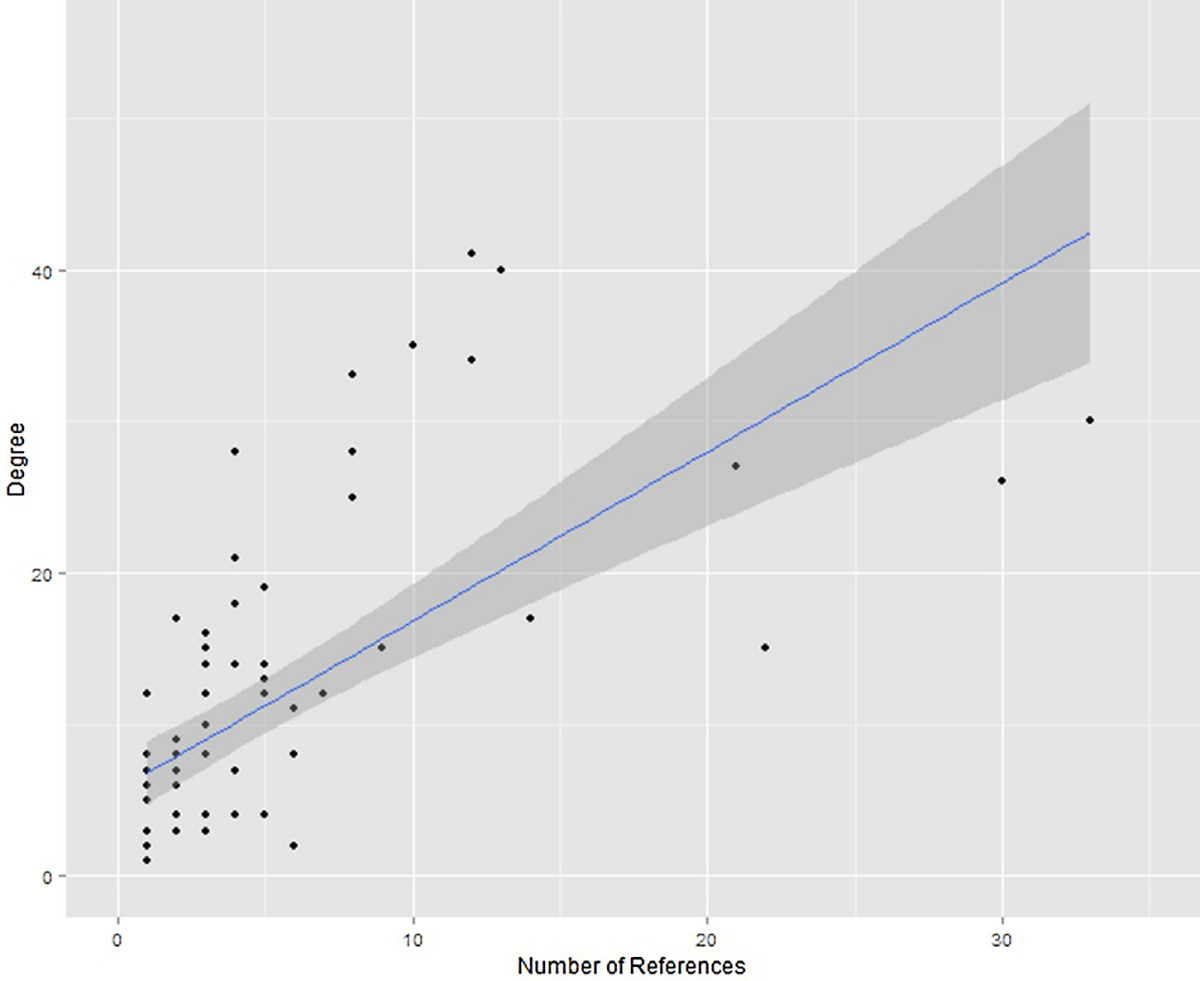
Correlation between degree (vertical axis) and number of references (horizontal axis) for nematode parasites of wild ungulate species (black dots). Blue line is fitted linear model, and gray area shows standard error.

In wild species, degree is highly correlated with the number of database references which mention that particular species (Fig. 1, Spearman's rank correlation, rho = 0.82, *p* < 0.001).

Figure 1 shows that degree increases with number of references. While some wild host species have a high degree with few references, it is likely that including those hosts with low degree and low number of references would bias the result. Consequently, although all host–parasite associations were included for the purpose of determining host-specificity, results are only reported for hosts with degree greater than 10.

**Fig. 2:**
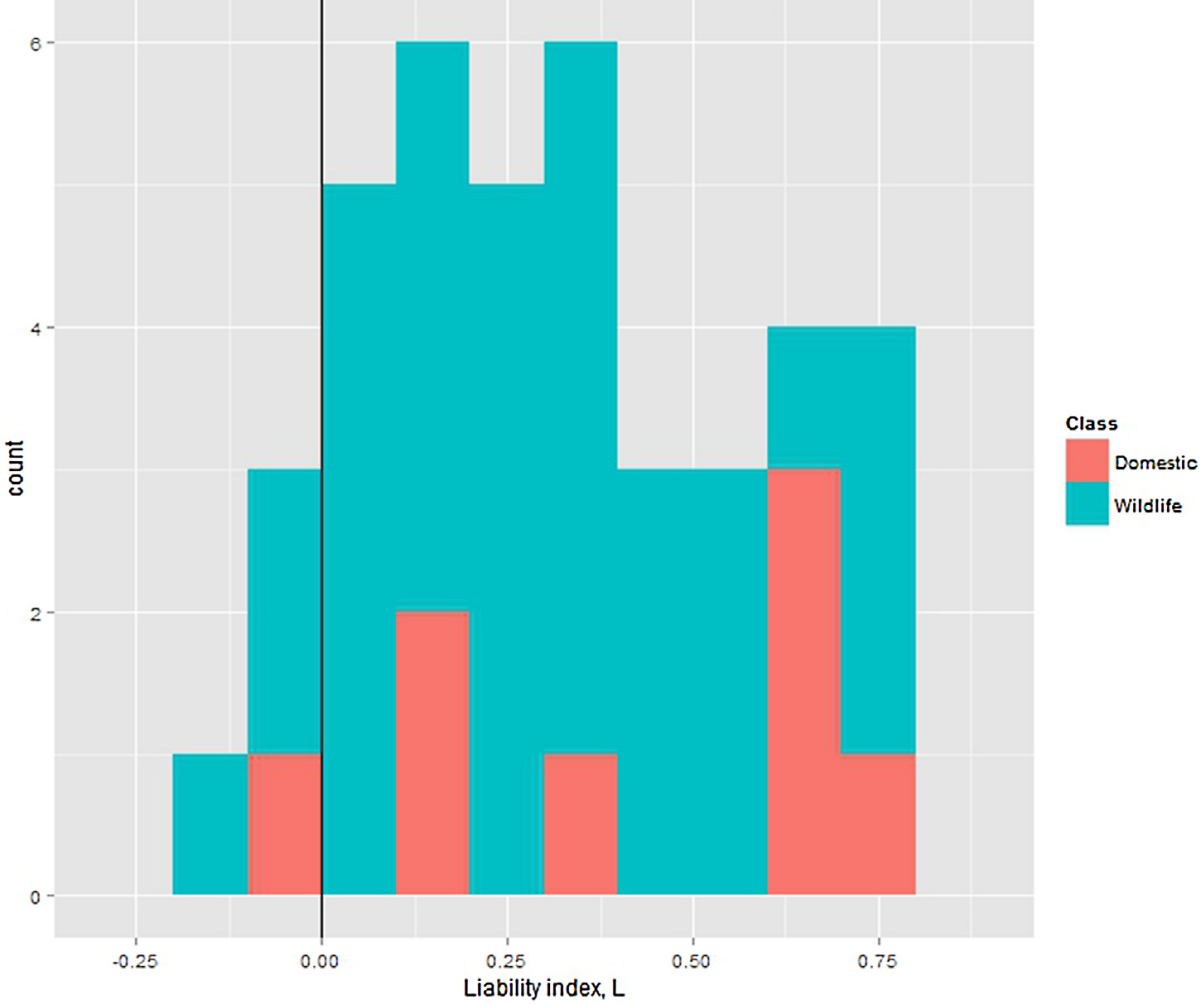
Histogram of liability index, *L*, for domestic and wild species with degree greater than 10.

All the wild and domestic host species examined share some of their parasites with hosts in the other category (Table 3 and  Table 4). Host-specific parasites make up less than half of the nematode parasites infecting any of the ungulate host species. Goats, sheep, donkeys, and camelids are the most liable of domestic animals to nematodes carried by wildlife, in terms of the wildlife-associated parasites found in their nematode fauna, while horses are the least liable. Of the wildlife species, alpine ibex, plains zebra, mouflon, mountain zebra, and Spanish ibex are the most liable to nematodes carried by domestic animals, while white-tailed deer, wild boar, grey rhebok, sika deer, and common warthog are the least liable. In general, the number of parasites shared between wild and domestic species is high: 18–76% of parasitic nematode species found in wild hosts are also found to infect domestic hosts, and 42–77% of parasitic nematode species found in domestic hosts are also found to infect wild hosts. Most of the hosts have a sharing index above zero, indicating that more parasites are shared with the other category than are unique (Fig. 2).

### 3.3 Specific cases in the literature

The available literature on transmission of nematodes between wildlife and livestock is limited, although this issue has been studied since the 1930s or earlier (Mönnig, 1931). A few local or regional reviews have examined the topic from a broad multi-host and multi-parasite perspective, while many focus on a particular pair of closely related host species. From these papers, it is not clear what the overall risk of transmission is between wildlife and livestock because the results are different for different systems and different regions, as might be expected. In this section, we describe case studies of nematode overlap between wild and domestic ungulates, before going on to consider emerging general patterns and issues for further study. The cases presented here, while not directly comparable due to differences in methodology and taxonomic resolution, demonstrate many of the challenges, limitations, and gaps in existing knowledge of studies of cross-boundary transmission of parasites.

An early review of the possibility of helminth transmission between wildlife and livestock in Botswana concluded that although some transmission is likely to occur, it probably does not contribute to clinical disease in domestic animals (Carmichael, 1972). A comprehensive review and synthesis of gastrointestinal strongyle nematodes in wild and domestic hosts in North America concludes that although the parasite fauna in wild ruminants is only partially segregated from that of domestic ruminants, a “relatively low percentage of the entire fauna is shared among these host groups,” and cross-transmission is not common in that region (Hoberg et al., 2001). On the other hand, a review of helminth fauna, primarily nematodes, in Mongolian domestic and wild ruminants found that 62% (67/108) of parasite species were present in both wild and domestic hosts, while only 27% (29/108) were host specific (Sharhuu and Sharkhuu, 2004).

Two more recent papers have used discriminant analysis to assess the degree of overlap between host–parasite communities. First, a study of abomasal nematodes of Italian alpine ruminants found that parasite communities could be distinguished by discriminant analysis between all species except for mouflon and domestic sheep (Zaffaroni et al., 2000). The study concludes that host specific parasites tend to dominate the communities of their principal hosts, while generalist parasites such as *Haemonchus contortus, Trichostrongylus axei* and *T. capricola* are found in many host species but at intermediate abundances. Second, a synthesis of studies of equid parasites in southern Africa found that helminth community structure was significantly different between plains zebra, mountain zebra, horses, and donkeys, although seven species were shared by all four host species and horses and donkeys shared most helminth species between them (Matthee et al., 2004). These studies both show that there is overlap in the parasite species shared between different hosts, but the consequences of these parasite communities and their components to each host species are not well understood.

On the other hand, due to the known detrimental impact of lungworm in cattle, deer, and other ungulates, several studies have examined the possibility of *Dictyocaulus* spp. transmission between wild and domestic hosts (Johnson et al., 2003). In the United States, an attempt to experimentally infect cattle with lungworm (*Dictyocaulus viviparus*) from white-tailed deer (*Odocoileus virginianus*) was not successful (Bates et al., 2000). Wapiti (elk, *Cervus canadensis*) in the United States have been experimentally infected with *D. viviparus* from cattle, though it was not highly pathogenic (Foreyt et al., 2000). Wapiti in Alberta, Canada, were examined for diseases that also infect cattle, because they share rangeland. Cattle lungworms, *D. viviparus*, were found in 32% of the wapiti examined (Kingscote et al., 1987). In New Zealand, *Dictyocaulus* species from separate cattle and red deer (*Cervus elaphus*) populations were experimentally cross-transmitted to parasite-free cattle and red deer (Johnson et al., 2003). In the study, the authors assume that lungworms taken from deer were *D. eckerti*, and that lungworms taken from cattle were *D. viviparus*. However, analysis of ribosomal DNA indicates that the phylogeny of *Dictyocaulus* species is not clearly parallel to host phylogeny (Höglund et al., 2003), and the genetic relationship between *Dictyocaulus* species from different hosts is an active area of research (Gasser et al., 2012). In New Zealand, transmission was successful from cattle to deer, though the number of adult parasites in the lungs at necropsy and shedding of larvae in feces were much higher in the deer infected with lungworms from deer. No adult lungworms were found in the cattle infected with lungworms from deer at necropsy, though intermittent shedding of larvae did occur, indicating that the cattle were susceptible but then able to eliminate the infection (Johnson et al., 2003). In France, the prevalence of *D. eckerti* in roe deer (*Capreolus capreolus*) was found to be correlated with land cover and not with density of roe deer or that of domestic ruminants (Hugonnet and Cabaret, 1987). In Argentina, the sheep lungworm *D. filaria* was found in wild guanacos in a reserve adjacent to a sheep ranch, and guanacos showed signs of lung congestion on necropsy (Beldomenico et al., 2003).

Another factor which has driven research into nematode transmission across the wildlife–domestic boundary is the potential for spillover into humans, with domestic species acting as a link between humans and wild animals. Few nematodes are reported in the literature that infect wild and domestic ungulates as well as humans. For example, *Trichinella* is a well-studied zoonotic nematode with a complex life cycle involving domestic and wild carnivores, domestic pigs, and wild boars. *Trichinella spiralis* is normally associated with domestic pigs, but is found in wild European boars in areas where domestic pigs are raised, and has also been found in bushpigs and warthogs in Africa. Other species (*T. britovi, T. nativa, T. pseudospiralis, T. papuae*) are found in domestic pigs but cannot be maintained in a domestic habitat without the presence of wild hosts. *Trichinella* species have also been detected in domestic sheep, cows, reindeer, roe deer, and horses. Outbreaks in humans tend to occur in areas where pigs are raised using traditional practices or graze in wild areas (Pozio, 2000; Pozio et al., 2009). Another zoonotic nematode reported in the literature to be carried by wild and domestic ungulates is *Oesophagostomum bifurcum*. This species is found in livestock including cattle, sheep, goats, and pigs, and wildlife such as red deer, roe deer, and moose (Böhm et al., 2007). It has only been found to infect humans in west Africa, and it is not understood why transmission to humans appears to occur here but not elsewhere (Pit et al., 1999).

In many parts of the world wildlife species have been examined for evidence of infection by nematode species normally associated with domestic species, without also reporting whether those species are concurrently found in domestic hosts in the area. In some cases, the authors are primarily interested in the consequences for conservation of wild species, while other papers are concerned about spillover into domestic animals. A few studies have used experimental infection to demonstrate the potential for transmission between host species.

In North America (Ontario, Canada), moose (*Alces alces*) killed by hunters in an agricultural area were examined to determine the role of other species in their parasite composition. Five nematode species were found, but only whipworm species (*Trichuris discolor* and *T. ovis*) were also found in livestock and were likely to have been transmitted from them (Hoeve et al., 1988). A recent study on genetic markers in whipworms supports the possibility of transmission of whipworms between different ruminant species, because samples from ruminants clustered together phylogenetically (Hansen et al., 2013).

In Iran, wild boar (*Sus scrofa*) were examined for parasites and 16 species of helminth were collected, including 10 species of nematode. The species found were compared to known records for parasites in domestic animals: *Oesophogastomum turkestanicum* is shared with domestic and wild ruminants, and *Ascarops strongylina, Physocephalus sexalatus, Ascaris suum, Strongyloides ransomi, Oesophagostomum dentatum, Trichuris suis* and *Metastrongylus apri* are shared with domestic pigs (Eslami and Farsad-Hamdi, 1992).

In Argentina, guanacos in a game reserve adjacent to a sheep ranch were found to be infected with *Marshallagia* and *Nematodirus* species, as well as *D. filaria*. The authors concluded that these parasites were likely to have spilled over from sheep as camelid-specific species of these genera were not known (Beldomenico et al., 2003).

Several studies have looked at nematodes of wild ungulates in southern Africa in relation to domestic species. In Zambia, lechwe antelope (*Kobus leche kafuensis*) had several species of nematode which also infect domestic species, including *Marshallagia marshalli, D. filaria, Cooperia punctata*, and *Oesophagostomum columbianum*. These lechwe were in contact with domestic animals in a wetland area, and the authors concluded that this is likely to lead to bi-directional transmission of parasites between lechwe and domestic cattle, though no evidence of clinical impacts of the nematodes were found in the lechwe (Phiri et al., 2011). In Uganda, impala freely graze and browse with livestock (Ankole cattle and goats) in areas adjacent to a national park. Comparisons of average worm burden (eggs per gram) in impala inside and outside the national park were done by a t-test, and no significant difference was found (Ocaido et al., 1999). It was not clear from the study that the samples from inside and outside the park were from different groups of impala, as groups of female impala are not territorial and travel to find resources (Estes, 1991). In the study, the species found in impala were only identified to genus level, due to limitations on the specific identification of nematode eggs. It was therefore inconclusive whether any transmission was occurring – to answer this would require specific information on whether impala and livestock are carrying the same parasite strains, as well as a controlled study comparing animals with and without domestic contact. In another study in Uganda, fecal samples from impala, buffalo, zebra, cattle, and goats in a mixed grazing system were classified to genus level, and 22 types of helminth were shared (Ocaido et al., 2004). Literature from South Africa supports the overlap between nematodes infecting wild and domestic species in South Africa but does not directly address transmission. Experimental infection has also been used to demonstrate that wild African antelope harbor parasites that can be transmitted to domestic animals. A study from 1931 in South Africa experimentally infected *Haemonchus contortus, Oesophagostomum columbianum, Haemonchus bedfordi*, and *Cooperia* spp. from wild antelope to sheep (Mönnig, 1931).

In the United Kingdom, deer species have been studied to determine the risk of transmission of nematodes and other parasites to domestic animals. Culled red deer (*Cervus elaphus*) and sika deer (*Cervus nippon*) in Scotland were examined and parasites identified to the genus level (Böhm et al., 2006). A further study reviewed parasites, including helminths, in deer in the UK in terms of the risk to domestic animals and humans. Multi-host systems were suggested to contribute to persistence of parasites and make control more difficult (Böhm et al., 2007). The potential for deer (fallow deer, red deer, and roe deer) to carry and spread anthelmintic-resistant abomasal nematodes was recently tested experimentally (Chintoan-Uta et al., 2014). Anthelmintic-resistant *Haemonchus contortus* were found in free-living, untreated roe deer and successfully experimentally transmitted to sheep and cattle, indicating that deer could play a role in the spread of anthelmintic resistance in the UK. Additionally, a study which genotyped *H. contortus* in the Italian Alps found that parasite strains were well mixed between wild and domestic ungulate hosts, suggesting that regular transmission between species occurs (Cerutti et al., 2010). In northern Europe, some reindeer populations are semi-domestic and others are wild. Domestic reindeer in Finland and Sweden have been examined to determine whether microfilariae of *Setaria* species are transmitted to moose or roe deer. Moose do not carry the same strains as reindeer, but roe deer could spread the parasite between different populations as they travel long distances when migrating (Laaksonen et al., 2009, 2010).

In Kazakhstan, the migration of saiga (*Saiga tatarica tatarica*) is also likely to contribute to nematode infection in domestic sheep. Saiga and sheep were studied using *post mortem* examination: of 31 nematode species found, 29 have been found in domestic livestock, though there was no evidence that helminths adversely affected the saigas (Morgan et al., 2005). A model of saiga migration predicts transmission of *Marshallagia* from sheep to saigas in the winter and back to sheep in the summer, and of *Haemonchus* from sheep to saigas in the summer and back to sheep in the winter (Morgan et al., 2005, 2006, 2007). In this system, saigas seem unlikely to act as long-term reservoirs of nematode infection for livestock, but could act to translocate parasites between domestic populations.

The studies stated earlier suggest that many generalist nematodes are found in both wild and domestic species. They also indicate that methods such as experimental transmission and genetic analysis can be used to provide evidence for host-switching which descriptive parasitological methods cannot prove. However, due to the inherent limitations of observational data, the small number of studies, and the broad diversity of nematodes, hosts, and ecosystems examined, measuring the total impact of multi-host nematodes on wildlife conservation, on domestic animal production, and on ecosystem health is not straightforward.

## 4 Discussion and synthesis

Parasitic nematodes that infect ungulates are diverse and often generalist. Most wild and domestic species have few host-specific parasites, which make up less than half of the total number of nematode parasite species found in a given host. Among domestic species, goats, sheep, donkeys, and camelids have a high liability index, with values greater than 0.6. Camels have an intermediate but positive liability index that still indicates that many more species are shared across categories than are host-specific. On the other hand, horses, pigs, and cattle have similar numbers of host-specific nematodes as generalists shared with wildlife. These domestic species are all well studied with a large number of nematode species (greater than 25) reported to be associated with them.

Few wild species have been as extensively studied as domestic species, and the maximum number of references found for nematodes in a particular wild species is 33 for red deer. Many of the wild species assessed had five or fewer references. The number of references describing the nematode fauna of a given species is significantly correlated with the number of parasitic nematodes listed. This is as expected because large sample sizes will be required to find rare parasites. A curve showing the total number of parasites as the number of references increases can be created and used to determine whether the number of parasites asymptotes (Matthee et al., 2004). In the present study, we used a cut off for inclusion of 10 parasites per host based on comparison of the number of references with degree (number of parasite species) for all hosts, rather than including species with a set number of references, because references varied in extensiveness and methods. That is, because some studies examined one particular host–parasite relationship, while others tried to classify all parasites within a host, using a reference-degree curve for each host might not accurately show whether the sampling effort was sufficient to reach an asymptote.

For wild ungulates, like domestic species, host-specific parasites constitute fewer than half the total nematode fauna, and several species (plains zebra, mountain zebra, mouflon, and alpine ibex) share more than 70% of their parasites with domestic animals. The parasite fauna of mouflon have previously been found to be indistinguishable from the parasite fauna of domestic sheep by discriminant analysis (Zaffaroni et al., 2000). These four species are all sympatric with closely related domestic species (donkey, sheep, goats), and the mouflon is thought to be one of the ancestors of domestic sheep. It is expected that closely related hosts are likely to have similar parasite assemblages, and use of phylogenies of hosts and parasites is a well-established method to study evolution and speciation (Brooks and McLennan, 2002; Hafner et al., 1994). In addition, host species in the same guild and with similar resource utilization patterns will provide opportunities for host-switching that could lead to convergence of parasite faunas (Hoberg and Brooks, 2008).

In this review, we did not have sufficient data to compare the abundance of different nematode species within a host. Zaffaroni and colleagues found that in the alps, host-specific parasites dominate the communities of their hosts, while generalist parasites tend to occur at lower abundances (Zaffaroni et al., 2000). Further studies of the contribution of shared parasite species to total parasite burden rather than only species richness would be a step toward understanding the impact that generalist parasites have on a host.

Few studies look explicitly at the implications of shared parasites for management in wild or domestic species. When there are strong seasonal patterns of contact with wildlife contributing to parasite burden in livestock, such as with saiga and sheep in Kazakhstan, this information could be used to seasonally target anthelmintic treatment in domestic animals (Morgan et al., 2006). In addition, while anthelmintic-resistant parasites may be picked up and spread by wild hosts, these hosts may also carry an untreated refugia population of parasites which will help to slow the evolution of resistance (Chintoan-Uta et al., 2014). Another possible benefit of cross-species transmission is the potential for cross-immunity between individual host-associated strains with different levels of impact on the host, such as has been suggested for *Dictyocaulus* in roe deer and cattle (Divina and Höglund, 2002). In order to develop appropriate management strategies, it will be necessary to understand the role that each host plays in the system, such as acting as a reservoir versus a spillover host. A theoretical framework for characterizing systems with reservoir hosts has recently been developed (Viana et al., 2014). It will be useful to extend this framework to examine transmission of multi-host nematodes and other macroparasites.

One of the primary limitations of the literature on parasitic nematodes of wild and domestic ungulates is a geographic bias whereby 84% of the references found for this review were from Africa, Europe, and North America. Geographical differences in species composition and the different uses of and reliance on livestock mean that conclusions from these three regions are not necessarily comparable or valid for other regions of the world. Any management decisions would be specific to a particular economic context. However, there may be some large-scale patterns that can be assumed to hold true, such as the high number of generalist nematodes and the universal potential for transmission across the wildlife–livestock boundary. Based on existing information, we can conclude that generalist parasites contribute more than specialists to the diversity of parasites in both wild and domestic species.

Actual transmission of nematodes between wildlife and livestock is not guaranteed by the same parasite species being present in multiple hosts. Transmission will depend on the relative abundances of the host species as well as on the degree to which there is contact or shared grazing areas (Morgan et al., 2004). As a result, the generalism of parasite species described here is indicative of the realized niche of the parasite. Future changes in host distributions and mosaic faunas due to climate change, human land-use change will lead to additional opportunities for previously undescribed host–nematode interactions as well as the general risk of emerging infectious diseases (Agosta et al., 2010; Altizer et al., 2013).

In addition, the studies described here generally rely on morphological identification of parasite species. It is a difficult challenge to prove that transmission is occurring between different host populations, rather than showing that they share morphologically identical parasites, as the same parasite species may have separately circulating strains. In addition, cryptic species will be mis-identified when morphological identification alone is used (Hoberg et al., 1999). In order to address the problem of misidentification, voucher specimens of parasites collected should be archived centrally, such as in the United States National Parasite Collection (Lichtenfels et al., 1992). As new technologies are developed, these specimens could be used to validate identifications and contribute to a better understanding of parasite ecology beyond what may be found in a single study.

Molecular phylogeography is beginning to be used to improve our understanding of parasite distribution and biology (Morgan et al., 2012), and progress could accelerate with new genomic tools and resources. The use of genetic markers to identify the degree of overlap between populations of generalist parasites in different host species, such as has been done with *H. contortus* (Cerutti et al., 2010) and *Dictyocaulus* (Höglund et al., 2003), would go a long way to improve our understanding of multi-host/multi-parasite systems. Greater use of controlled intervention studies, in which treatment of one population is shown to alter the nematode fauna of in-contact host species, would also be illuminating. Finally, the implications of cross-transmission for ungulate health, fitness/production, and the spread and control of diseases of importance to conservation and agricultural production deserve more consideration if studies of nematode overlap are to have direct relevance to current societal challenges.

In this paper we developed a simple framework by which to compare the liability of wild and domestic animals to cross-boundary transmission of parasites. Although there are many limitations in existing data, an integrated approach to studies of parasites of wild and domestic animals will assist in developing predictions of the impact of changes in contact between wild and domestic species as people, livestock, and wildlife come under increasing pressure due to population growth and climate change.

## Figures and Tables

**Table 1:** Number of studies by GEO-3 region. Sums to 242 because one reference described studies from two regions.

Region	Number of references
Polar	13
Asia and Pacific	20
Europe	76
Latin America and Caribbean	6
North America	60
West Asia (Middle East)	0
Africa	67

**Table 2:** References by multi-host or multi-parasite classification.

	Single host	Multiple hosts	
Single parasite	67	23	**90 (37%)**
Multiple parasites	102	49	**151 (63%)**
	**169 (70%)**	**72 (30%)**	**241**

**Table 3:** Number of nematode species reported in a range of wild ungulates, and extent of overlap with domestic ungulates.

Host	Degree	Unique	*P_unique_*	Shared	*P*_shared_	RefNum	*L*
Alpine ibex	16	0	0	12	0.75	3	0.75
Plains zebra	35	1	0.029	26	0.74	10	0.71
Mouflon	21	1	0.048	16	0.76	4	0.71
Mountain zebra	33	2	0.061	24	0.73	8	0.67
Spanish ibex	17	1	0.059	11	0.65	2	0.59
Chamois	41	3	0.073	25	0.61	12	0.54
Hartebeest	12	1	0.083	7	0.58	5	0.50
Mule deer	15	1	0.067	8	0.53	9	0.47
Red duiker	18	0	0	8	0.44	4	0.44
Southern reedbuck	14	0	0	6	0.43	3	0.43
European bison	28	3	0.11	14	0.50	4	0.39
Common duiker	18	1	0.056	8	0.44	4	0.39
Fallow deer	19	1	0.053	8	0.42	5	0.37
Tsessebe	15	0	0	5	0.33	3	0.33
Saiga	12	3	0.25	7	0.58	1	0.33
Bontebok	14	1	0.071	5	0.36	3	0.29
Moose	15	3	0.20	7	0.47	22	0.27
Gemsbok	25	0	0	6	0.24	8	0.24
Greater kudu	28	3	0.11	9	0.32	8	0.21
Roe deer	40	7	0.18	15	0.38	13	0.20
Bushbuck	11	0	0	2	0.18	6	0.18
Impala	34	3	0.088	9	0.26	12	0.18
Mountain reedbuck	12	1	0.083	3	0.25	3	0.17
Springbok	21	2	0.095	5	0.24	4	0.14
Nyala	14	2	0.14	3	0.21	5	0.071
Red deer	30	7	0.23	9	0.30	33	0.067
Reindeer	17	4	0.24	5	0.29	14	0.059
White-tailed deer	26	9	0.35	10	0.38	30	0.038
Wild boar	27	13	0.48	13	0.48	21	0
Grey rhebok	13	3	0.23	2	0.15	5	−0.077
Sika deer	12	6	0.50	5	0.42	7	−0.083
Common warthog	14	4	0.29	2	0.14	4	−0.14

Wild ungulate species with degree greater than 10 are included. Degree (number of parasite species); Unique (number of parasite species not found in any other host); *P_unique_* (proportion of total that are unique); Shared (number of parasites shared with domestic ungulates); P_shared_ (proportion of total shared with domestic ungulates); RefNum (number of references from GMPD describing that host species); *L*, the liability index. *P_unique_* and *P*_shared_ do not sum to 1 because parasite species shared with other wild species are not included.

**Table 4:** Number of nematode species found in domestic ungulates, and extent of overlap with wild ungulates.

Host	Degree	Unique	*P*_unique_	Shared (Wild)	*P*_shared_	L
Goat	40	1	0.025	29	0.73	0.70
Sheep	42	2	0.048	29	0.69	0.64
Donkey	33	1	0.030	22	0.67	0.64
Camelid (Llama and alpaca)	26	4	0.15	20	0.77	0.62
Camel	47	13	0.28	31	0.66	0.38
Cattle (inc. *Bos indicus* and *B. taurus*)	50	18	0.36	26	0.52	0.16
Pig	27	12	0.44	15	0.56	0.11
Horse	72	31	0.43	30	0.42	−0.014

Degree (number of parasite species); Unique (number of parasite species not found in any other host); *P_unique_* (proportion of total that are unique); Shared (number of parasites shared with wild ungulates); *P*_shared_ (proportion of total shared with wild ungulates); *L*, the liability index. *P_unique_* and *P*_shared_ do not sum to 1 because parasite species shared with other domestic species are not included.
